# Efficacy of COVID-19 control measures on post-vaccination outbreak in Italian Long Term Care Facilities: implications for policies

**DOI:** 10.3389/fpubh.2023.1091974

**Published:** 2023-06-06

**Authors:** Alba Malara, Marianna Noale, Caterina Trevisan, Angela Marie Abbatecola, Gilda Borselli, Carmine Cafariello, Pietro Gareri, Stefano Fumagalli, Enrico Mossello, Stefano Volpato, Fabio Monzani, Alessandra Coin, Chukwuma Okoye, Giuseppe Bellelli, Stefania Del Signore, Gianluca Zia, Raffaele Antonelli Incalzi, Annapina Palmieri, Giorgio Fedele, Graziano Onder, The GeroCovid Vax Working Group

**Affiliations:** ^1^Associazione Nazionale Strutture Territoriali-Humanitas Foundation, Rome, Italy; ^2^Neuroscience Institute, National Research Council, Padua, Italy; ^3^Department of Medical Sciences, University of Ferrara, Ferrara, Italy; ^4^Department of Medicine, University of Padua, Padua, Italy; ^5^Alzheimer's Disease Day Clinic, Azienda Sanitaria Locale, Frosinone, Italy; ^6^Italian Society of Gerontology and Geriatrics, Florence, Italy; ^7^Long Term Care Clinic, Provincia Romana dei Camilliani, Rome, Italy; ^8^Center for Cognitive Disorders and Dementia (CDCD) Catanzaro Lido – ASP Catanzaro, Catanzaro, Italy; ^9^Department of Experimental and Clinical Medicine, University of Florence and Division of Geriatric and Intensive Care Medicine, Azienda Ospedaliero-Universitaria Careggi, Florence, Italy; ^10^Geriatrics Unit, Department of Clinical and Experimental Medicine, University of Pisa, Pisa, Italy; ^11^School of Medicine and Surgery, University of Milano-Bicocca and Acute Geriatric Unit, San Gerardo Hospital, Monza, Italy; ^12^Bluecompanion Ltd., Londra, United Kingdom; ^13^Unit of Geriatrics, Department of Medicine, Campus Bio-Medico University and Teaching Hospital, Rome, Italy; ^14^Department of Cardiovascular, Endocrine-Metabolic Diseases and Aging, Istituto Superiore di Sanità, Rome, Italy; ^15^Department of Infectious Disease, Istituto Superiore di Sanità, Rome, Italy; ^16^Department of Geriatrics, Universita' Cattolica Sacro Cuore, Rome, Italy; ^17^Fondazione Policlinico Gemelli Istituto di Ricovero e Cura a Carattere Scientifico, Rome, Italy

**Keywords:** Long Term Care Facilities (LTCFS), outbreak control measures, COVID-19 vaccination, SARS CoV-2 infection, pandemic fatigue

## Abstract

**Background:**

Numerous individual and organizational factors can influence the spread of SARS-CoV-2 infection in Long Term Care Facilities (LTCFs). A range of outbreak control measures are still implemented in most facilities involving administrations, staff, residents and their families. This study aims to evaluate which measure could influence the transmission of SARS-CoV-2 infection among residents during the period March 2021-June 2022.

**Methods:**

We enrolled 3,272 residents aged ≥60 years. The outbreak control measures adopted to prevent or manage the infection included entry regulations, contact-regulating procedures, and virological surveillance of residents and staff. The association between LTCFs' and participants' characteristics with new cases of COVID-19 infections was analyzed using multilevel logistic regression models.

**Results:**

In 33.8% of the facilities 261 cases of SARS-CoV-2 infection were reported. Among participant characteristics, gender and age were not associated with SARS-CoV-2 infection, while having received the vaccine booster dose was protective against infection [Odds Ratio (OR) = 0.34, 95% Confidence Interval (CI) 0.12–0.99, *p* = 0.048]. In addition, the implementation of protected areas for family visits was associated with a significant reduction of the probability of infections (OR = 0.18, 95% CI 0.03–0.98, *p* = 0.047). Overall, about 66% of the variability in the probability of SARS-CoV-2 infection during the observational period may be due to facility structure characteristics and 34% to the participant characteristics.

**Conclusions:**

These data showed that vaccination booster doses and family visit restriction-control are still needed to make the LTCFs safer against SARS-CoV-2 infection.

## Introduction

Following the devastating impact of the first wave of COVID-19 on Long Term Care (LTC) system, LTC Facilities (LTCFs) have made an extraordinary effort to provide safer care for older people. Indeed, residents of LTCFs are at an extremely high risk of developing severe complications following a SARS-CoV-2 infection due to numerous reasons, including comorbidities, immune senescence, and reliance on care from others in a community setting (1, 2). In August 2021, the pandemic resumed in Europe due to the emergence of the Omicron variant of SARS-CoV-2 that changed the landscape of the pandemic drastically with a surge in new cases even among vaccinated individuals, including those living in LTCFs (1–3). The risk of outbreak in LTCFs depends upon a series of variables such as, the level of vaccination coverage of the residents, the duration of vaccine coverage, the vaccination rates for staff working in the sector, the use of individual protection devices, visitation restriction, quarantine, and others (4, 5). Previous studies highlighted that a range of anti-contagion measures are frequently implemented in most facilities involving administrations, staff, residents and their families, but the evidence remains uncertain (6). For this reason, it is problematic to identify the effects of the measures to reduce the development of new SARS-CoV-2 cases in LTCFs. This study aims to evaluate which measures could influence the transmission of SARS-CoV2 infection among residents.

## Methods

We included 77 Italian LTCFs which are part of GeroCovid VAX network: GeroCovid Vax is a multicenter study promoted by is a multicenter study promoted by the Italian Society of Gerontology and Geriatrics (SIGG) (Florence, Italy), the Italian National Institute of Health (Istituto Superiore di Sanità—ISS, Rome, Italy), and sponsored by the Italian Medicines Agency (Agenzia Italiana del Farmaco—AIFA). Specific details relating to the study as well as, on the sampling methodology are reported in previous reports (7, 8). For this study, 3,272 residents (aged ≥60 years, with an estimated life expectancy and an expected stay in LTCFs ≥3 months) were identified between February and May 2021 and followed for 12 months. For each participant, we collected data on demographic characteristics, lifestyle, chronic diseases, and clinical outcomes in an electronic registry (8). Of the 3,272 residents, 2,670 (81.6%) completed the follow-up and were included in the present analysis. The mean age of the sample was 83.2 ± 9.1 years and 72.5% were female; all participants had received a primary vaccine cycle (two doses of the mRNA anti-SARS-CoV-2 vaccine, either mRNA-1273 or BNT162b2; at least one dose if they had been affected by previous infection) and 1,895 of these (90.6%) received a third dose of an mRNA vaccine (either mRNA-1273 or BNT162b2) between 6 and 9 months from the first vaccine dose. Regarding the clinical course of SARS CoV-2 positive residents, it was defined as asymptomatic (no symptoms), paucisymptomatic (only one symptom) and symptomatic (more than one symptom). The outbreak control measures used to prevent or manage the infection included entry regulation measures to prevent residents, staff or visitors from introducing the virus into the facility. These measures included quarantine for newly admitted residents in COVID-19 isolation areas, testing new admissions for infection, entry restrictions and preventing visitors from entering facilities, as well as suspending non-urgent medical visits outside the facility. In Italy, the quarantine (from 10 to 15 days) and the type of swab (molecular-pcr test and rapid antigen-test) for newly admitted residents was regulated by decrees of the Ministry of Health, considering the contagion index of the various Italian regions, the number of vaccination doses of the new patient and the vaccination rate of facilities (9). The contact-regulating and transmission-reducing measures to prevent viral transmission included wearing masks or other PPE (gloves, disposable gowns, booties) for the staff and visitors, extra cleaning, and protected areas for family visits. The association between LTCFs' and participants' characteristics, and new cases of SARS-CoV-2 infection during the follow-up (March 2021–June 2022) was analyzed using multilevel logistic regression models, with LTCFs at the first level and participants at the second level. As described in Ene et al. (10), three consecutive models were defined: the first included no predictors, only random effect for the intercept; the second also included first level variables (LTCFs characteristics) as fixed effects; the third included the first level (LTCFs' characteristics) and the second level variables (participants' characteristics) as fixed effects. The best fitting model was identified by conducting a deviance test. Intraclass correlation coefficient (ICC) was computed to evaluate how much of the total variation in the probability of the outcome is accounted for by LTCFs' and participants' characteristics (10). All statistical tests were two-tailed and statistical significance was assumed for *p*-value < 0.05. The analyses were performed using SAS, V.9.4 (SAS Institute, Cary, NC).

## Results

The main characteristics of the 77 participating facilities and of the 2,670 residents enrolled in the study are described in Table 1. The use of personal protective equipment (PPE), the restriction of visits, the suspension of non-essential specialized procedures, the implementation of environmental sanitation interventions, quarantine and COVID-19 isolation areas were adopted in most facilities. Staff and residents' virological surveillance protocols were common, and the 88.8% of the facilities had a staff vaccination rate between 75 and 100%. During the monitored pandemic period (July 2021–June 2022), 26 facilities (33.8%) 261 cases of SARS-CoV-2 infection were reported. The outbreaks were more frequent in northern regions of Italy with respect to central and southern regions (57.7 vs. 42.3%, respectively; *p* = 0.003), and in the medium/large sized LTCFs (≥50 beds) with respect to small (<50 beds) (76.9 vs. 23.1%, respectively; *p* = 0.037). Overall, 171 of the 261 residents with SARS-CoV-2 infection (65.5%) had an asymptomatic or paucisymptomatic clinical course and were managed in the facilities, and only 10 were transferred to the hospital. Among individual variables, sex and age were not associated with the onset of SARS-CoV-2 infection during the observed pandemic wave, while having received the vaccine booster dose was a protective factor against infection [Odds Ratio (OR) = 0.34, 95% Confidence Interval (CI) 0.12–0.99, *p* = 0.048] (Table 2). Among LTCFs features, the implementation of protected areas for family visits was significantly associated with a reduced probability of infection (OR = 0.18, 95% CI 0.03–0.98, *p* = 0.047) (Table 2). Overall, about 66% of the variability in the probability of SARS-CoV-2 infection during the period may be attributable to characteristics of facility structures and 34% to characteristics of the participants.

**Table 1:** Main characteristics of the subjects and the LTCFs participating into the study.

	**Overall**	**IV pandemic wave** ^*^ **, SARS-CoV-2 infections**		**P-value**
		**No**	**Yes**	
**Subjects' level**, ***n***	2,670	2,409	261	
Age, years, mean ± SD	83.2 ± 9.1	83.2 ± 9.1	83.5 ± 9.0	0.595
Sex, females, *n* (%)	1,936 (72.5)	1,744 (72.4)	192 (73.6)	0.688
Booster, *n* (%)	1,895 (90.6)	1,691 (90.8)	204 (89.1)	0.410
Asymptomatic or paucisymptomatic, *n* (%)	171 (6.4)	–	171 (65.5)	–
**LTCFs' level**, ***n***	77	51	26	
Site type, *n* (%)				0.471
Nursing home	42 (54.6)	29 (56.9)	13 (50.0)	
Medicalized nursing home	14 (18.2)	10 (19.6)	4 (15.4)	
Assisted living	12 (15.6)	6 (11.8)	6 (23.1)	
Retirement home	5 (6.4)	2 (3.9)	3 (11.5)	
Other (follow-up rehab or specialized Alzheimer's unit)	4 (5.2)	4 (7.8)	0 (0.0)	
**Size**, ***n*** **(%)**				
Small (< 50 beds)	29 (37.7)	23 (45.1)	6 (23.1)	0.037
Medium (50–150 beds)	41 (53.3)	26 (51.0)	15 (57.7)	
Large (>150 beds)	7 (9.0)	2 (3.9)	5 (19.2)	
**Italian geographical areas**				
Northern	25 (32.5)	10 (19.6)	15 (57.7)	0.003
Central	21 (27.3)	17 (33.3)	4 (15.4)	
Southern	31 (40.2)	24 (47.1)	7 (26.9)	
**LRCFs' professionals**				
General practitioner, *n* (%)	30 (39.0)	20 (39.2)	10 (38.5)	0.949
Physician, *n* (%)	59 (76.6)	37 (72.6)	22 (84.6)	0.237
Geriatrician, *n* (%)	41 (53.3)	30 (58.8)	11 (42.3)	0.170
Professional nurse, *n* (%)	77 (100.0)	51 (100.0)	25 (100.0)	–
Physiotherapist, *n* (%)	73 (94.8)	48 (94.1)	25 (96.2)	1.000
Social worker, *n* (%)	55 (71.4)	36 (70.6)	19 (73.1)	0.819
Professional educator, *n* (%)	53 (68.8)	34 (66.7)	19 (73.1)	0.566
Psychologist, *n* (%)	52 (67.5)	34 (66.7)	18 (69.2)	0.820
Medical manager, *n* (%)	48 (63.2)	31 (62.0)	17 (65.4)	0.772
Vaccination rates for the staff, *n* (%)				0.180
<25%	3 (3.9)	3 (5.9)	0 (0.0)	
25–49%	1 (1.3)	0 (0.0)	1 (3.9)	
50–75%	5 (6.5)	2 (3.9)	3 (11.5)	
75–100%	68 (88.3)	46 (90.2)	22 (84.6)	
Use of individual protection devices, *n* (%)	77 (100)			
Surgical masks	60 (77.9)	39 (76.5)	21 (80.8)	0.667
FFP2/FFP3 masks	69 (89.6)	44 (86.3)	25 (96.2)	0.254
Gloves	77 (100.0)	51 (100.0)	26 (100.0)	–
Disposable gowns	69 (89.6)	46 (90.2)	23 (88.5)	1.000
Booties	57 (74.0)	40 (78.4)	17 (65.4)	0.217
COVID-19 isolation areas, *n* (%)	75 (97.4)	49 (96.1)	26 (100.0)	0.547
Staff regular testing, *n* (%)	75 (98.7)	50 (98.0)	25 (100.0)	1.000
Resident regular testing, *n* (%)	67 (88.2)	43 (86.0)	25 (92.3)	0.710
Entry Restrictions, *n* (%)				0.082
No	0 (0.0)	0 (0.0)	0 (0.0)	
Partial	17 (22.1)	8 (15.7)	9 (34.6)	
Total	60 (77.9)	43 (84.3)	17 (65.4)	
Suspension of external medical visits, *n* (%)	31 (41.3)	18 (36.0)	13 (52.0)	0.185
Extraordinary sanitation procedures, *n* (%)	75 (97.4)	50 (98.0)	25 (96.2)	1.000
Family visit with 48 h negative swab, *n* (%)	13 (17.1)	9 (17.7)	4 (16.0)	1.000
Family entry with PPE (without swab), *n* (%)	7 (9.2)	4 (7.8)	3 (12.0)	0.678
Family entry not allowed, *n* (%)	18 (23.7)	10 (19.6)	8 (32.0)	0.233
Protected areas for family visits, *n* (%)	49 (63.6)	37 (72.6)	12 (46.2)	0.023

^*^Period July 2021–June 2022.

**Table 2:** Two-level (LTCFs; residents) generalized linear dichotomous models with outcome SARS-CoV-2 infections during the IV pandemic wave.

	**Model 1 (only intercept)**	**Model 2 (intercept + participants' characteristics)**	**Model 3^a^ (intercept + participants' + LTCFs characteristics)**	**Model 3 (intercept** + **participants'** + **LTCFs characteristics)**		
				**OR**	**95% CI**	**P-value**
**Fixed effects**						
Intercept (SE)	−4.60^*^ (0.57)	−3.49^*^ (1.11)	−9.10^*^ (3.65)			
**Participants' characteristics**						
Female vs Males		0.10 (0.21)	0.11 (0.21)	1.12	0.74–1.70	0.590
Age, year		−0.01 (0.01)	−0.01 (0.01)	1.00	0.98–1.02	0.949
Booster		−1.15^*^ (0.50)	−1.07^*^ (0.54)	0.34	0.12–0.99	0.048
**LTCFs' characteristics**						
Vaccination rates for the LTCF's staff >90%			2.21 (1.39)	9.07	0.58–141.0	0.115
Use of surgical masks			−0.39 (0.91)	0.68	0.11–4.02	0.668
Use of FFP2/FFP3 masks			3.16 (1.72)	23.6	0.80–693.6	0.097
Use of disposable gowns			0.88 (1.38)	2.41	0.16–36.1	0.525
Use of booties			0.60 (1.04)	1.83	0.24–14.2	0.563
Staff virology surveillance			1.45 (1.68)	4.28	0.16–116.1	0.388
Suspension of external medical visits			−0.05 (0.87)	0.95	0.17–5.27	0.951
Extraordinary environments' sanitization			−0.56 (2.41)	0.57	0.01–64.3	0.817
Family visit with 48 h negative swab			1.16 (1.10)	3.19	0.37–27.3	0.290
Family visit with PPE (without swab)			−0.87 (1.31)	0.42	0.03–5.46	0.507
Family visit prohibited			0.73 (1.07)	2.08	0.26–16.8	0.494
Protected areas for family visits			−1.70^*^ (0.86)	0.18	0.03–0.98	0.047
**Error variance**						
Level-2 intercept	6.46^*^ (2.42)	7.46^*^ (2.97)	3.47^*^ (1.51)			
**Model fit**						
−2LL	1,287.32	1,031.52	947.53			

^*^p < 0.05; ^a^best fitting model; ICC = 0.663 (~66.3% of the variability in the COVID-19 infections rate during the IV pandemic wave, in our study, is accounted for by the LTCFs characteristics, and 33.7% of the variability is accounted for by the residents' characteristics or other unknown factors).

## Discussion

To protect residents and staff of LTCFs from COVID-19, various protective measures have been recommended by several national and international guidelines (11, 12), which have been implemented to different levels over the last 2 years to reduce the risk of outbreaks and super-spread events in LTCFs. However, the clinical features of residents as well as, the frequent atypical or asymptomatic manifestations of COVID-19 in these populations (13–15) have contributed to the spread of infection in this context. Our results support the concept that the booster dose vaccination and availability of protected areas for family visits can prevent SARS-CoV-2 infections and their consequences in frail and complex older adults living in LTCFs. This study, based on real-world data, showed that a range of anti-contagion measures are frequently implemented in most facilities involving administrations, staff, residents and their families. For this reason, it is problematic to identify the respective effects of individual measures on the outcome. These measures may prevent SARS-CoV-2, but they do not exclude the possibility of new outbreaks that may impact the wellbeing of residents. Selected measures were associated with a decreased incidence of SARS-CoV-2 infections, while certain non-pharmacologic measures have also been hypothesized to negatively impact the mental and physical health of residents by reducing individual wellbeing with an increased the risk of depression and anxiety (16, 17), especially in those with dementia (18). Conversely, other authors have pointed out that vaccine coverage might lead to a false perception of safety from infection, resulting in additional behavioral changes that undermine the residents' safety. Indeed, studies highlight that changes in protective non-pharmaceutical interventions (NPIs) adherence over time can substantially alter the population-level effect of the vaccine on morbidity and mortality in LTCFs. Love et al. (19) built a stochastic model to simulate outbreaks in LTCF populations with different vaccine coverage and NPIs adherence to evaluate their interaction effects. They concluded that vaccination combined with strong adherence to the NPIs resulted in the lowest morbidity and mortality in LTCFs population. Vaccinating health care workers improved outcomes in unvaccinated LTCFs residents but had lower impact when the NPI adherence declined (19). Another critical factor that can complicate efforts to contain COVID-19 is the so called “pandemic fatigue”. This phenomenon, characterized by demotivation to follow recommended NPIs, such as mask use and social distancing and other, could be implicated as a factor in new peaks of infection in LTCFs (20). In this context, it is important to clarify which measures must be maintained in the next future.

One limitation to our study is due to low power (0.20). Thus, results should be considered as research orienting and such findings need to be confirmed with longer follow-ups as well as, in additional LTC population samples. However, strengths of this present study include its real-life nature, the large number of residents involved and the length of follow up. These findings provide an important basis for future studies.

In conclusion, our findings show that vaccination booster doses and visit control-restriction are the most effective preventive measures. Future research is needed to design a care model providing safety for frail LTCF residents and perceived sustainable by residents, families, and care teams.

## Data availability statement

The raw data supporting the conclusions of this article will be made available by the authors, without undue reservation.

## Ethics statement

The studies involving human participants were reviewed and approved by the Ethical Committee at the Spallanzani Institute (permission no. 264/2021; 26 January 2021; Rome, Italy) and the local Ethics Committees at the participating sites. The patients/participants provided their written informed consent to participate in this study.

## Author contributions

AM, GO, and RA: study concept and design. GeroCovid Vax Working Group and GBo: acquisition of data. MN and AM: analysis and interpretation of data. AM, GO, RA, MN, and CT: drafting of the manuscript. GO, RA, AM, MN, PG, AA, CC, SF, EM, CT, SV, AC, GBe, FM, CO, SD, and GZ: critical revision of the manuscript for important intellectual content. All authors contributed to the article and approved the submitted version.

## GeroCovid Vax Working Group

Angela Marie Abbatecola [ASL Frosinone; in collaborazione con RSA INI Città Bianca, Veroli (FR); RSA San Germano, Piedimonte San Germano (FR); RSA Santa Maria, Castrocielo (FR); RSA Villa degli Ulivi, Sant'Elia Fiumerapido (FR); RSA Villa Letizia, Patrica (FR)]; Domenico Andrieri [RSA Villa Santo Stefano, S. Stefano di Rogliano (CS); RSA Villa Silvia, Altilia Grimaldi (CS)]; Raffaele Antonelli Incalzi [Università Campus Bio-Medico, Roma]; Francesca Arenare [ASP Golgi Redaelli, Istituto Piero Redaelli, Milano]; Viviana Bagalà [CRA Capatti, Riva del Po (FE); CRA Mantovani, Copparo (FE); CRA Plattis, Cento (FE); CRA Quisisana, Ostellato (FE); CRA Ripagrande, ASP Ferrara]; Tatjana Baldovin [Università di Padova]; Riccardo Bernardi [RSA Estensiva per DCCG, IHG, Guidonia (RM)]; Alessandra Bianchi [RSA Arici Sega, Fondazione Brescia Solidale, Brescia]; Paola Bianchi [Associazione Nazionale Strutture Territoriali e per la Terza Età, Roma]; Raffaella Bisceglia [RSA Universo Salute Opera Don Uva, Foggia]; Ivan Bissoli [RSA Villa San Giuseppe, Crotone]; Fabio Bontempi [ASP Golgi Redaelli, Istituto Piero Redaelli, Milano]; Gilda Borselli [Società Italiana di Gerontologia e Geriatria, Firenze]; Luigi Bottaro [ASP Golgi Redaelli, Istituto Piero Redaelli, Milano]; Elisa Bottoni [Centro Benessere (Riabilitazione), Frosinone]; Silvia Brandi [Casa di Riposo Torriglia, Chiavari (GE)]; Claudio Bravin [RSA Fondazione Casa Industria, Brescia]; Maria Adele Buizza [Azienda Speciale Casa di Riposo M. Bonincontri, Brescia]; Carmine Cafariello [RSA Disabili Alto Mantenimento, IHG, Guidonia (RM); RSA Estensiva per DCCG, IHG, Guidonia (RM); RSA Estensiva, IHG, Guidonia (RM); RSA Geriatria Alto Mantenimento, IHG, Guidonia (RM); RSA Geriatria Alto Mantenimento, IHG, Guidonia (RM); RSA Intensivo, IHG, Guidonia (RM); RSA Villa Sacra Famiglia, IHG, Roma]; Alessia Maria Calabrese [RSA Bastia, Livorno; RSA Casa Mimosa, Pisa; RSA Coteto, Livorno; RSA Madonna della Fiducia, Calambrone (PI); RSA Pio Istituto Campana, Seravezza (LU); RSA Villa Isabella, Pisa]; Valeria Calsolaro [RSA Bastia, Livorno; RSA Casa Mimosa, Pisa; RSA Coteto, Livorno; RSA Madonna della Fiducia, Calambrone (PI); RSA Pio Istituto Campana, Seravezza (LU); RSA Villa Isabella, Pisa]; Marta Canepa [RSA Villa Sorriso, Rapallo (GE)]; Carla Capasso [RSA Fondazione Casa Industria, Brescia]; Mariagrazia Capuano [RSA Gianà, Qualiano (NA)]; Sebastiano Capurso [RSA Bellosguardo, Civitavecchia (RM)]; Gabriele Carbone [RSA Estensiva per DCCG, IHG, Guidonia (RM)]; Marialudovica Carducci [RSA Villa Sacra Famiglia, IHG, Roma]; Silvia Carino [Casa di Riposo San Domenico, Lamezia Terme (CZ); Casa Protetta Madonna del Rosario, Lamezia Terme (CZ); Centro di Riabilitazione San Domenico, Lamezia Terme (CZ); RSA San Domenico, Lamezia Terme (CZ); RSA Villa Elisabetta, Cortale (CZ)]; Nicoletta Cattaneo [RSA Sandro Pertini, ASST Rhodense, Garbagnate Milanese (MI)]; Francesco Ceravolo [Casa Protetta San Domenico, Palermiti (CZ); RSA Santa Maria del Monte, Petrizzi (CZ)]; Maria Angelica Dorotea Chiesara [RSA San Pietro, Monza]; Danila Clerici [RSA Sandro Pertini, ASST Rhodense, Garbagnate Milanese (MI)]; Pierpaolo Clerici [ASST Ovest Milanese, Legnano (MI)]; Alessandra Coin [Azienda Ospedale Università Padova]; Vieri Collacchioni [RSA Nuova Villa Rio, San Godenzo (FI)]; Mauro Colombo [ASP Golgi Redaelli, Istituto Piero Redaelli, Milano]; Michela Compiano [RSA Le due Palme, Sestri Levante (GE)]; Giuseppina Costanza [RSA Villa delle Palme, Villafrati (PA)]; Giovanna Crupi [RSA2 Sindromi da immobilizzazione, ASP Palermo]; Roberta Cucunato [RSA Villa Santo Stefano, S. Stefano di Rogliano (CS); RSA Villa Silvia, Altilia Grimaldi (CS)]; Manuela Marina D'Abramo [RSA L'Ulivo, Fondazione Cittadella della Carità, Taranto]; Emilia D'Agostino [Casa Protetta San Giuseppe, San Sosti (CS)]; Ferdinando D'Amico [RSA San Giovanni di Dio, Patti (ME); RSA Sant'Angelo di Brolo (ME)]; Antonio De Simone [RSA La Quiete, Castiglione Cosetino (CS)]; Stefania Del Vecchio [RSA Bastia, Livorno; RSA Casa Mimosa, Pisa; RSA Coteto, Livorno; RSA Madonna della Fiducia, Calambrone (PI); RSA Pio Istituto Campana, Seravezza (LU); RSA Villa Isabella, Pisa]; Maria Deleo [RSA Villa delle Palme, Villafrati (PA)]; Annalaura Dell'Armi [RSA Geriatria Alto Mantenimento, IHG, Guidonia (RM)]; Tommasina Di Brango [RSA Santa Maria, Castrocielo (FR)]; Anna Di Lonardo [Istituto Superiore di Sanità, Roma]; Maria Raffaella Di Nanno [RSA Il Sorriso, Sanità Più, Foggia; RSA Universo Salute Opera Don Uva, Foggia]; Babette Dijk [RSA Estensiva, ASL4, Chiavari (GE)]; Luisa Elmo [RSA Villa Sorriso, Marano sul Panaro (MO)]; Giorgio Fedele [Istituto Superiore di Sanità, Roma]; Marisa Ferraro [RSA Il Sorriso, Sanità Più, Foggia]; Christian Ferro [RSA Sant'Angelo di Brolo (ME)]; Claudia Fiorucci [RSA Estensiva, IHG, Guidonia (RM); RSA Intensivo, IHG, Guidonia (RM)]; Francesca Fortunato [Università di Foggia]; Pasquale Froncillo [RSA Dimora Marigold, Pozzuoli (NA)]; Domenico Galasso [Casa Protetta San Domenico, Palermiti (CZ); RSA Santa Maria del Monte, Petrizzi (CZ)]; Nicola Galdiero [RSA Gianà, Qualiano (NA)]; Caterina Galdiero [RSA Gianà, Qualiano (NA)]; Stefania Gallo [RSA Sandro Pertini, ASST Rhodense, Garbagnate Milanese (MI)]; Pier Paolo Gasbarri [Associazione Nazionale Strutture Territoriali e per la Terza Età, Roma]; Maria Grazia Gennai [RSA Nuova Villa Rio, San Godenzo (FI)]; Giuliana Ghiselli Ricci [RSA Villa Sorriso, Marano sul Panaro (MO)]; Elisa Giribaldi [Casa di Riposo Torriglia, Chiavari (GE)]; Carmen Godeanu [CRA Villa dei Ciliegi, Valsamoggia (BO)]; Samuele Gommaraschi [RSA Villaggio Amico, Gerenzano (VA)]; Roberta Granata [RSA Villa Sacra Famiglia, IHG, Roma]; Giada Ida Greco [RSA La Quiete, Castiglione Cosetino (CS)]; Angela Greco [RSA2 Sindromi da immobilizzazione, ASP Palermo]; Antonio Grillo [ASP Golgi Redaelli, Istituto Piero Redaelli, Milano]; Gianbattista Guerrini [RSA Arici Sega, Fondazione Brescia Solidale, Brescia]; Mauro Guglielmo [RSA Sandro Pertini, ASST Rhodense, Garbagnate Milanese (MI)]; Labjona Haxhiaj [RSA AltaVita, Istituzioni Riunite di Assistenza, Padova]; Claudio Giuseppe Iacovella [RSA Santa Maria, Castrocielo (FR)]; Marina Indino [RSA Villaggio Amico, Gerenzano (VA)]; Valerio Alex Ippolito [Casa Protetta Villa Azzurra, Roseto Capo Spulico (CS)]; David Kanah [ASP Golgi Redaelli, Istituto Piero Redaelli, Milano]; Liudmila Kountsevich [RSA Disabili Alto Mantenimento, IHG, Guidonia (RM)]; Jovan Leci [CRA Villa Teresa, Sasso Marconi (BO)]; Federica Limongi [Istituto di Neuroscienze - Sezione Invecchiamento, CNR, Padova]; Agata Lipari [Casa di Riposo Domus Aurea, Africo (RC); Casa Protetta Universo, Africo (RC); RSA Universo, Africo (RC)]; Vincenzo Longo [Casa di Riposo Domus Aurea, Africo (RC); Casa Protetta Universo, Africo (RC); RSA Universo, Africo (RC)]; Stefania Maggi [Consiglio Nazionale delle Ricerche, Padova]; Alba Malara [Casa di Riposo San Domenico, Lamezia Terme (CZ); Casa di Riposo Villa Marinella, Amantea (CS); Casa Protetta Madonna del Rosario, Lamezia Terme (CZ); Casa Protetta Villa Azzurra, Roseto Capo Spulico (CS); Centro di Riabilitazione San Domenico, Lamezia Terme (CZ); RSA Casa Amica, Fossato Serralta (CZ); RSA La Quiete, Castiglione Cosetino (CS); RSA San Domenico, Lamezia Terme (CZ); RSA Villa Elisabetta, Cortale (CZ); RSA Villa Santo Stefano, S. Stefano di Rogliano (CS); RSA Villa Silvia, Altilia Grimaldi (CS)]; Leonarda Maltese [RSA Bastia, Livorno; RSA Casa Mimosa, Pisa; RSA Coteto, Livorno; RSA Madonna della Fiducia, Calambrone (PI); RSA Pio Istituto Campana, Seravezza (LU); RSA Villa Isabella, Pisa]; Maria Marotta [RSA Villa Letizia, IHG, Patrica (FR)]; Giuseppe Mazzarella [RSA Villa degli Ulivi, Sant'Elia Fiumerapido (FR)]; Hior Melnik [Casa di Riposo Domus Aurea, Africo (RC); Casa Protetta Universo, Africo (RC); RSA Universo, Africo (RC)]; Pasquale Minchella [Azienda Ospedaliera Pugliese Ciaccio, Catanzaro]; Paolo Moneti [RSA Villa Gisella, Firenze]; Fabio Monzani [RSA Bastia, Livorno; RSA Casa Mimosa, Pisa; RSA Coteto, Livorno; RSA Madonna della Fiducia, Calambrone (PI); RSA Pio Istituto Campana, Seravezza (LU); RSA Villa Isabella, Pisa]; Walter Morandotti [ASP Golgi Redaelli, Istituto Piero Redaelli, Milano]; Francesco Morelli [RSA Villa San Giuseppe, Crotone]; Maria Grazia Mortola [RSA Villa Sorriso, Rapallo (GE)]; Marianna Noale [Istituto di Neuroscienze - Sezione Invecchiamento, CNR, Padova]; Chukwuma Okoye [RSA Bastia, Livorno; RSA Casa Mimosa, Pisa; RSA Coteto, Livorno; RSA Madonna della Fiducia, Calambrone (PI); RSA Pio Istituto Campana, Seravezza (LU); RSA Villa Isabella, Pisa]; Graziano Onder [Istituto Superiore di Sanità, Roma]; Patrizia Orlanducci [CRA Villa dei Ciliegi, Valsamoggia (BO); CRA Villa Teresa, Sasso Marconi (BO)]; Barbara Paganelli [RSA Bosco in Città, Brugherio (MB); RSA Scaccabarozzi, Ornago (MB)]; Michele Pagano [RSA Buon Pastore Onlus, Palermo]; Nicola Pagano [RSA Quadrifoglio, Giugliano in Campania (NA)]; Raffaele Palladino [RSA Il Sorriso, Sanità Più, Foggia; RSA Universo Salute Opera Don Uva, Foggia]; Annapina Palmieri [Istituto Superiore di Sanità, Roma]; Magda Palumeri [RSA Buon Pastore Onlus, Palermo]; Simone Paolini [RSA AltaVita, Istituzioni Riunite di Assistenza, Padova; RSA Opera Immacolata Concezione, Padova]; Raimondo Paternò [Casa di Riposo Domus Aurea, Africo (RC); Casa Protetta Universo, Africo (RC); RSA Universo, Africo (RC)]; Angela Pavan [ASP Golgi Redaelli, Istituto Piero Redaelli, Milano]; Loris Pelucchi [RSA Sandro Pertini, ASST Rhodense, Garbagnate Milanese (MI)]; Agostino Perri [RSA La Quiete, Castiglione Cosetino (CS)]; Francesco Perticone [Casa Protetta San Domenico, Palermiti (CZ); RSA Santa Maria del Monte, Petrizzi (CZ)]; Rosanna Pesce [RSA Quadrifoglio, Giugliano in Campania (NA)]; Sabrina Pigozzo [CRA di Cittadella (PD)]; Francesco Pili [Casa Protetta San Teodoro, Crotone; Casa Protetta Villa del Rosario, Crotone; RSA San Teodoro, Crotone; RSA Villa del Rosario, Crotone]; Rosa Prato [Università di Foggia]; Rosanna Pullia [RSA Bastia, Livorno; RSA Casa Mimosa, Pisa; RSA Coteto, Livorno; RSA Madonna della Fiducia, Calambrone (PI); RSA Pio Istituto Campana, Seravezza (LU); RSA Villa Isabella, Pisa]; Ahmad Amedeo Qasem [RSA Bastia, Livorno; RSA Casa Mimosa, Pisa; RSA Coteto, Livorno; RSA Madonna della Fiducia, Calambrone (PI); RSA Pio Istituto Campana, Seravezza (LU); RSA Villa Isabella, Pisa]; Francesco Raffaele Addamo [RSA San Giovanni di Dio, Patti (ME)]; Cecilia Raffaelli [RSA AltaVita, Istituzioni Riunite di Assistenza, Padova]; Vincenzo Restivo [Università di Palermo]; Michela Fernanda Rigon [RSA Opera Immacolata Concezione, Padova]; Franco Romagnoni [CRA Capatti, Riva del Po (FE); CRA Mantovani, Copparo (FE); CRA Plattis, Cento (FE); CRA Quisisana, Ostellato (FE); CRA Ripagrande, ASP Ferrara]; Carmine Romaniello [RSA INI Città Bianca, Veroli (FR)]; Valentina Romano [RSA Fondazione Casa Industria, Brescia]; Maria Cristina Ruberto [Casa Protetta San Giuseppe, San Sosti (CS)]; Marcello Russo [ASL Frosinone]; Bruno Sala [RSA Luigi Accorsi, Legnano (MI)]; Sara Sambo [CRA di Cittadella (PD); RSA AltaVita, Istituzioni Riunite di Assistenza, Padova]; Maria Concetta Sciurti [ASP Golgi Redaelli, Istituto Piero Redaelli, Milano]; Antonietta Scriva [Casa di Riposo Domus Aurea, Africo (RC); Casa Protetta Universo, Africo (RC); RSA Universo, Africo (RC)]; Luca Secchi [RSA Villa Sorriso, Marano sul Panaro (MO)]; Vincenzo Settembrini [Casa Protetta Villa Azzurra, Roseto Capo Spulico (CS)]; Federica Sirianni [Casa di Riposo Villa Marinella, Amantea (CS); RSA Villa Santo Stefano, S. Stefano di Rogliano (CS); RSA Villa Silvia, Altilia Grimaldi (CS)]; Deborah Spaccaferro [RSA Estensiva, IHG, Guidonia (RM); RSA Intensivo, IHG, Guidonia (RM)]; Fausto Spadea [RSA Casa Amica, Fossato Serralta (CZ)]; Manuela Stefanelli [RSA Villa Sacra Famiglia, IHG, Roma]; Paola Stefanelli [Istituto Superiore di Sanità, Roma]; Brunella Stelitano [Casa di Riposo Domus Aurea, Africo (RC); Casa Protetta Universo, Africo (RC); RSA Universo, Africo (RC)]; Stefania Stringhini [RSA Villaggio Amico, Gerenzano (VA)]; Andrea Tarsitano [RSA Medicalizzata San Raffaele, Castiglione Cosentino (CS); RSA San Raffaele, Castiglione Cosentino (CS)]; Camilla Terziotti [CRA di Cittadella (PD); RSA AltaVita, Istituzioni Riunite di Assistenza, Padova; RSA Opera Immacolata Concezione, Padova]; Caterina Trevisan [CRA di Cittadella (PD); RSA AltaVita, Istituzioni Riunite di Assistenza, Padova; RSA Opera Immacolata Concezione, Padova]; Rita Ursino [RSA Geriatria Alto Mantenimento, IHG, Guidonia (RM)]; Giovanni Veneziano [ASP di Palermo]; Maria Teresa Vigliotta [RSA San Germano, Piedimonte San Germano (FR)]; Marco Vignati [RSA Sandro Pertini, ASST Rhodense, Garbagnate Milanese (MI)]; Eva Vignola [RSA Villa Gisella, Firenze]; Enrico Virgilio [CRA di Cittadella (PD)]; Maria Visconti [RSA Gianà, Qualiano (NA)]; Stefano Volpato [Università di Ferrara]; Susanna Vozzi [RSA Villaggio Amico, Gerenzano (VA)]; Sabrina Zaccone [Casa Protetta San Domenico, Palermiti (CZ); RSA Santa Maria del Monte, Petrizzi (CZ).
